# Mortality and cardiorenal outcomes among heart failure patients with zinc deficiency: a multicenter retrospective cohort study of 8,290 patients

**DOI:** 10.3389/fnut.2025.1589907

**Published:** 2025-04-28

**Authors:** Yu-Min Lin, Wan-Ling Tu, Kuo-Chuan Hung, Tsung Yu, Jheng-Yan Wu, Chih-Cheng Lai

**Affiliations:** ^1^Division of Cardiology, Department of Internal Medicine, Chi Mei Medical Centre, Tainan, Taiwan; ^2^Department of Nutrition Therapy, E-Da Hospital, Kaohsiung, Taiwan; ^3^Department of Anesthesiology, Chi Mei Medical Center, Tainan, Taiwan; ^4^Department of Public Health, College of Medicine, National Cheng Kung University, Tainan, Taiwan; ^5^Department of Nutrition, Chi Mei Medical Center, Tainan, Taiwan; ^6^Department of Intensive Care Medicine, Chi Mei Medical Center, Tainan, Taiwan; ^7^School of Medicine, College of Medicine, National Sun Yat-sen University, Kaohsiung, Taiwan

**Keywords:** zinc deficiency, heart failure, major adverse kidney events, major adverse cardiovascular event, mortality

## Abstract

**Objective:**

This study aimed to examine the association between zinc deficiency (ZD) and the clinical outcomes in patients with heart failure (HF).

**Methods:**

This multicenter retrospective cohort study used the TriNetX network to identify adult patients with HF between January 1, 2010, and January 31, 2025. Patients with serum zinc levels below 70 μg/dL (ZD group) were propensity score-matched to those with levels between 70 and 120 μg/dL (control group) to minimize confounding. Primary outcomes included all-cause mortality, major adverse cardiovascular events (MACEs), and major adverse kidney events (MAKEs). Secondary outcome was all-cause hospitalization.

**Results:**

After matching, each group comprised 4,145 patients with well-balanced baseline characteristics. During the 1-year follow-up, the ZD group was associated with higher risks of all-cause mortality (hazard ratio [HR]: 1.46, 95% confidence interval [CI]: 1.29–1.66), MACEs (HR: 1.46, 95% CI: 1.30–1.64), and MAKEs (HR: 1.51, 95% CI: 1.34–1.70), as well as an higher risk of all-cause hospitalization (HR: 1.24, 95% CI: 1.16–1.32).

**Conclusion:**

Zinc deficiency in patients with HF is associated with increased risks of mortality, cardiovascular and kidney-related adverse events, and hospitalization. These findings highlight the potential clinical importance of zinc assessment and management in HF care.

## Introduction

Heart failure (HF) remains a leading cause of morbidity and mortality worldwide, exerting a substantial clinical and economic burden (1, 2). The prevalence in the general adult population is estimated to be 1–3%, with an incidence rate of approximately 1–20 cases per 1,000 individuals each year (3, 4). Moreover, HF-associated mortality has been rising annually, with a 5-year mortality rate ranging from 50 to 75% (3). The economic impact is similarly profound, with annual healthcare costs per person estimated at $11,000–$18,000 (3, 5).

Zinc is an essential trace element that plays a crucial role in immune function, oxidative stress regulation, and cardiovascular homeostasis (6–12). Previous reviews demonstrated that zinc deficiency (ZD) is highly prevalent among patients with HF, with serum zinc levels significantly lower compared to healthy controls (13, 14). Patients with HF often exhibit hyperzincuria due to the use of diuretics and renin-angiotensin-aldosterone system inhibitors, further exacerbating zinc depletion (15–19). Mechanistically, ZD may contribute to HF progression through multiple pathways, including increased oxidative stress, systemic inflammation, and endothelial dysfunction (20–23). An observational study found that ZD was associated with a worse prognosis in patients with HF (24). Additionally, preliminary results suggested that zinc supplementation may improve left ventricular ejection fraction (LVEF), highlighting its potential therapeutic role (25).

Despite these findings, large-scale studies investigating the impact of ZD on HF outcomes remain limited. In this multicenter study, we aimed to explore the association between ZD and HF outcomes, including all-cause mortality, major adverse cardiac events (MACEs), and major adverse kidney events (MAKEs).

## Methods

### Study design and database

The study used data from the TriNetX Analytics Network Platform, a comprehensive federated health research network encompassing electronic medical records (EMRs) from approximately 160 million patients across 140 healthcare organizations (HCOs) worldwide (26). This expansive dataset incorporates diverse variables, including demographic information, diagnoses, procedures, medications, laboratory results, genomic data, and HCO visit classifications. TriNetX facilitates secure, real-time access to de-identified, aggregated data derived from a geographically and ethnically diverse patient population, spanning hospitals, primary care facilities, and specialty treatment centers. The platform operates under a waiver from the Western Institutional Review Board, as it exclusively processes statistical summaries without accessing individual-level patient data. This investigation was conducted in adherence to the Strengthening the Reporting of Observational Studies in Epidemiology (STROBE) guidelines (27).

### Study population and definition of eligible patients

The study population consisted of adult patients with HF who underwent zinc testing within 1 year prior to their HF diagnosis between January 01, 2010, and January 31, 2025. The date of zinc testing was designated as the index date.

Study eligibility required participants to be 18 years or older with an HF diagnosis, identified using the International Classification of Diseases, Tenth Edition, Clinical Modification (ICD-10-CM) code I50. Participants were stratified into two groups based on their serum zinc levels: the zinc deficiency (ZD) group (serum zinc <70 μg/dL) and the control group (serum zinc 70–120 μg/dL) (28, 29). Serum zinc measurements were identified using Logical Observation Identifiers Names and Codes (LOINC) 5768-8 or 8245-3. To ensure sufficient clinical documentation, we included only patients with at least two EMRs within the observation window. To minimize protopathic and ascertainment bias, patients with any prior history of MACE or MAKE were excluded based on all available EMRs preceding the index date (Supplementary Table S1).

### Covariates

The selection of covariates was determined by their clinical significance, with particular focus on key comorbidities and risk factors that have established associations with mortality rates and cardio-renal outcomes. We evaluated baseline health parameters in accordance with contemporary medical understanding. For both study groups, we extracted data on baseline characteristics and covariates from the year preceding the index date, including demographic factors (age, sex, race), clinical parameters (estimated glomerular filtration rate [eGFR], albumin, Hemoglobin A1c [HbA1c], left ventricular ejection fraction [LVEF]), existing comorbidites, and prescribed medications.

The spectrum of evaluated comorbidities extended to cardiometabolic conditions (hypertension, hyperlipidemia, obesity), nutritional status (malnutrition), metabolic disorders (type 2 diabetes mellitus), substance use patterns (nicotine dependence, alcohol-related disorders), respiratory conditions (chronic lower respiratory diseases), hepatic function (liver diseases), renal status (chronic kidney disease), cardiovascular conditions (cerebrovascular diseases, atrial fibrillation and flutter, ischemic heart disease), autoimmune disorders (systemic lupus erythematosus), and malignancies (neoplasms). The medication profile analysis encompassed therapeutic agents for heart failure management (including angiotensin-converting enzyme inhibitors [ACEis], angiotensin receptor blockers [ARBs], beta-blockers, calcium-channel blockers, various classes of diuretics, and sodium-glucose cotransporter-2 inhibitors [SGLT2is]) and cholesterol management (statins) (Supplementary Table S2).

### Outcomes

The study evaluated both primary and secondary outcomes. Primary outcomes comprised all-cause mortality, MACEs, and MAKEs. The secondary outcome focused on all-cause hospitalization risk. MACEs encompassed acute myocardial infarction, stroke (including cerebral infarction and hemorrhage), ventricular arrhythmias (e.g., ventricular tachycardia, ventricular fibrillation), and cardiac arrest (30). MAKEs were characterized by end-stage kidney disease (ESKD), urgent dialysis initiation, or dialysis dependence (30). Patient follow-up commenced the day after the index date and continued until their final clinical visit, death, or one-year post-index date, whichever occurred first (Supplementary Table S3).

### Statistical analysis

For baseline characteristics, continuous variables were presented as means with standard deviations (SDs), while categorical variables were expressed as frequencies and percentages. To minimize selection bias and balance covariates between groups, we implemented propensity score matching (PSM) using a greedy nearest-neighbor algorithm, with a caliper width set at 0.1 pooled SDs. The effectiveness of matching was evaluated through standardized differences, where values less than 0.1 indicated adequate covariate balance (31).

After matching, we conducted survival analyses using the Kaplan–Meier method, with between-group differences assessed via the log-rank test. The association between zinc status and outcomes was quantified using Cox proportional hazards regression models to calculate hazard ratios (HRs). To evaluate the statistical significance of the differences between subgroups, we used a method that examined the CI overlap (32). Additionally, we calculated *E*-values to further gage the impact of potential unmeasured confounders on the primary and secondary outcomes (33). Statistical significance was defined as a two-sided *p* value below 0.05.

### Stratified analysis

We conducted stratified analyses to examine the heterogeneity of primary outcomes associations across multiple subgroups. These analyses included age stratification (18–64 versus ≥ 65 years), sex-specific differences (female versus male), and heart failure classification based on ejection fraction (heart failure with preserved ejection fraction [HFpEF], heart failure with mildly reduced ejection fraction [HFmrEF], and heart failure with reduced ejection fraction [HFrEF]). Additional stratification was performed according to clinical parameters, including the presence of malnutrition, overweight and obesity, renal function (eGFR < 45 versus ≥ 45 mL/min/1.73 m^2^), and glycemic control (HbA1c < 9% versus ≥ 9%).

### Sensitivity analysis

To evaluate the robustness of our findings, we conducted additional sensitivity analyses by modifying the definition of MACEs. In the first analysis, ventricular arrhythmias were excluded to reflect a more conventional MACE definition comprising only acute myocardial infarction, stroke, and cardiac arrest. In a second analysis, we expanded the MACE definition to include cardiomyopathy (ICD-10-CM: I42) to examine its impact as a clinically relevant cardiovascular complication in patients with HF. Additionally, we performed a new round of PSM that included the following variables: C-reactive protein (CRP), copper, selenium, prealbumin, transferrin, and urine albumin-to-creatinine ratio (UACR). These variables were selected to account for systemic inflammation, trace element status, and nutritional reserve. Outcomes were re-evaluated using Cox proportional hazards models.

## Results

### Study flow diagram

**Figure 1:**
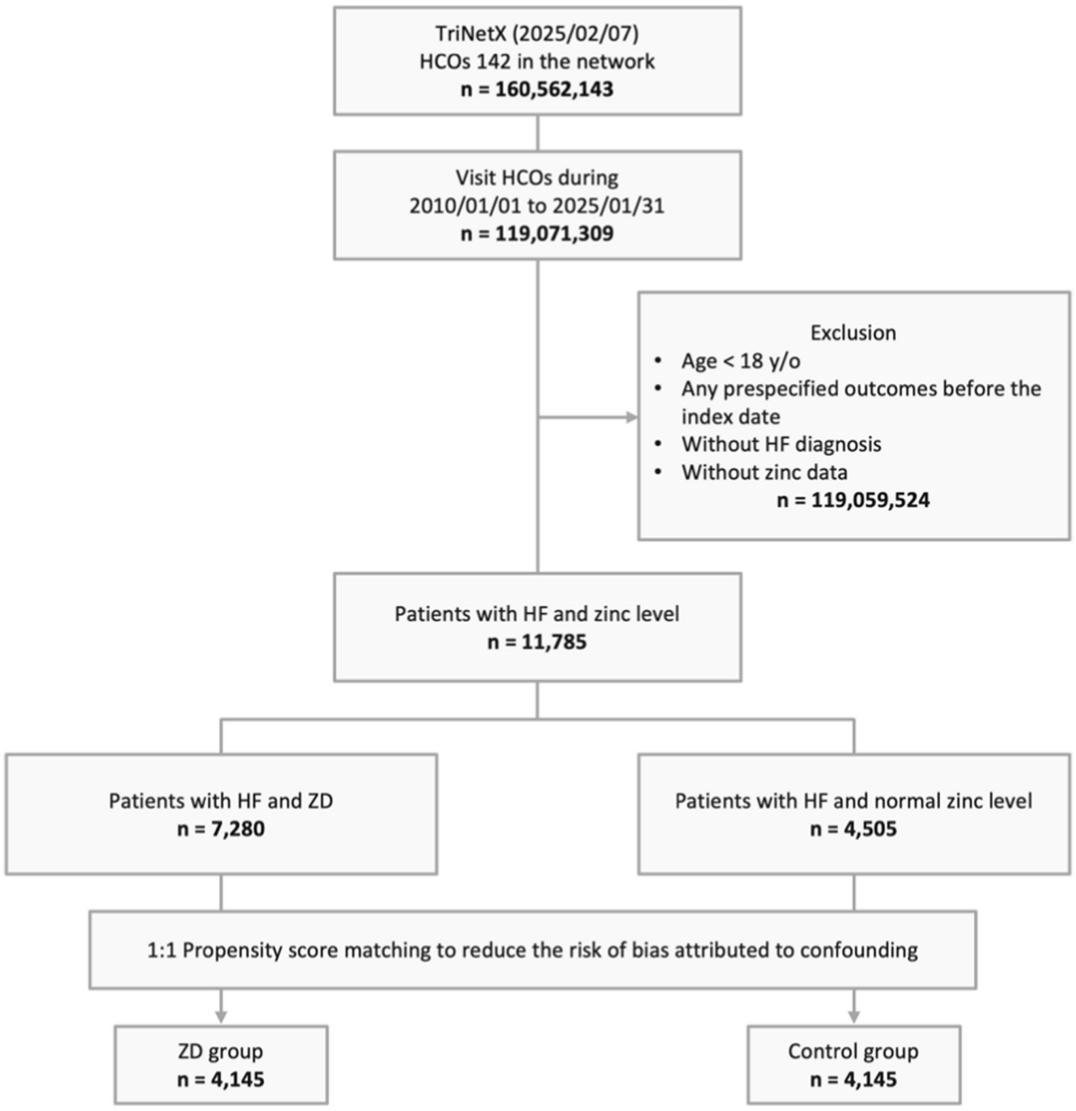
Study design and selection flow. HCO, healthcare organizations; HF, heart failure; y/o, years old; ZD, zinc deficiency.

From a total population of 160,562,143 patients across 142 HCOs in the TriNetX network, we identified 119,071,309 individuals with visits between January 01, 2010, and January 31, 2025. We excluded 119,059,524 patients who met one or more of the following criteria: age below 18 years, occurrence of prespecified outcomes before the index date, lack of zinc level measurements prior to the index date, or absence of HF diagnosis. Of the remaining 11,785 eligible patients with both HF and zinc measurements, 7,280 were categorized into the ZD group, while 4,505 comprised the control group with normal zinc levels. Following PSM, the final analysis included 4,145 patients in each group (Figure 1).

### Study population characteristics

Before PSM, there were significant differences in baseline characteristics between the ZD group (*n* = 7,280) and the control group (*n* = 4,505). Participants in the ZD group were older (66.2 ± 15.9 vs. 62.7 ± 14.4 years) and had a slightly lower proportion of female (54.5% vs. 56.9%). The ZD group had a higher prevalence of chronic conditions, including chronic kidney disease (32.1% vs. 23.6%), malnutrition (23.6% vs. 13.4%), and nicotine dependence (13.0% vs. 9.6%). Additionally, they had lower albumin levels (3.2 ± 0.7 vs. 3.7 ± 0.7 g/dL) and a higher proportion of patients with albumin ≤ 3.5 g/dL (67.8% vs. 46.6%). Use of medications such as diuretics (67.0% vs. 61.1%) and beta blockers (58.0% vs. 56.4%) was also more frequent in the ZD group (Table 1).

**Table 1:** Baseline characteristics of included subjects.

Variables	Before matching			After matching		
	ZD group (*n* = 7,280)	Control group (*n* = 4,505)	Std diff	ZD group (*n* = 4,145)	Control group (*n* = 4,145)	Std diff
Age at index, years						
Mean ± SD	66.2 ± 15.9	62.7 ± 14.4	0.215	63.5 ± 16.5	63.7 ± 16.2	0.009
Sex, n (%)						
Female	3,969 (54.5)	2,562 (56.9)	0.047	2,342 (56.5)	2,346 (56.6)	0.002
Male	3,307 (45.4)	1,940 (43.1)	0.048	1,799 (43.4)	1,797 (43.4)	0.001
Race, n (%)						
White	4,272 (58.7)	2,606 (57.9)	0.017	2,393 (57.7)	2,401 (57.9)	0.004
Black or African American	1,168 (16)	765 (17)	0.025	693 (16.7)	691 (16.7)	0.001
Asian	132 (1.8)	74 (1.6)	0.013	67 (1.6)	71 (1.7)	0.008
Other race	254 (3.5)	163 (3.6)	0.007	147 (3.6)	148 (3.6)	0.001
Unknown race	1,408 (19.3)	873 (19.4)	0.001	823 (19.9)	810 (19.5)	0.008
Estimated glomerular filtration rate, mL/min/1.73m^**2**^						
Mean ± SD	70.1 ± 39.4	72.8 ± 34.2	0.075	72.2 ± 36.7	71.9 ± 34.7	0.009
≤ 45, *n* (%)	2,934 (40.3)	1,315 (29.2)	0.235	1,304 (31.5)	1,296 (31.3)	0.004
Albumin, g/dL						
Mean ± SD	3.2 ± 0.7	3.7 ± 0.7	0.645	3.4 ± 0.7	3.6 ± 0.7	0.309
≤ 3.5, *n* (%)	4,938 (67.8)	2,097 (46.6)	0.440	2,089 (50.4)	2,093 (50.5)	0.002
HbA1c, %						
Mean ± SD	6.3 ± 1.6	6.4 ± 1.5	0.058	6.3 ± 1.5	6.4 ± 1.5	0.074
≥ 9, *n* (%)	434 (6)	282 (6.3)	0.012	240 (5.8)	254 (6.1)	0.014
Left ventricular ejection fraction, %						
Mean ± SD	54.7 ± 14.8	53.9 ± 14.7	0.050	55.0 ± 15.1	53.8 ± 14.8	0.076
< 40, *n* (%)	184 (2.5)	112 (2.5)	0.003	102 (2.5)	105 (2.5)	0.005
Comorbidities, *n* (%)						
Hypertension	4,474 (61.5)	2,867 (63.6)	0.045	2,593 (62.6)	2,614 (63.1)	0.010
Hyperlipidemia	3,521 (48.4)	2,367 (52.5)	0.084	2,117 (51.1)	2,115 (51.0)	0.001
Overweight and obesity	2,510 (34.5)	1,904 (42.3)	0.161	1,657 (40.0)	1,671 (40.3)	0.007
Malnutrition	1,715 (23.6)	603 (13.4)	0.264	610 (14.7)	600 (14.5)	0.007
Type 2 diabetes mellitus	2,369 (32.5)	1,373 (30.5)	0.044	1,279 (30.9)	1,281 (30.9)	0.001
Nicotine dependence	945 (13.0)	432 (9.6)	0.107	410 (9.9)	419 (10.1)	0.007
Alcohol related disorders	576 (7.9)	217 (4.8)	0.127	225 (5.4)	216 (5.2)	0.010
Chronic lower respiratory diseases	2,543 (34.9)	1,520 (33.7)	0.025	1,399 (33.8)	1,410 (34.0)	0.006
Diseases of liver	1,686 (23.2)	804 (17.9)	0.132	766 (18.5)	763 (18.4)	0.002
Chronic kidney disease	2,339 (32.1)	1,065 (23.6)	0.190	1,054 (25.4)	1,040 (25.1)	0.008
Cerebrovascular diseases	764 (10.5)	372 (8.3)	0.077	352 (8.5)	357 (8.6)	0.004
Atrial fibrillation and flutter	2,314 (31.8)	1,221 (27.1)	0.103	1,173 (28.3)	1,163 (28.1)	0.005
Ischemic heart diseases	2,896 (39.8)	1,694 (37.6)	0.045	1,573 (38)	1,575 (38)	0.001
Systemic lupus erythematosus	145 (2.0)	84 (1.9)	0.009	84 (2.0)	83 (2.0)	0.002
Neoplasms	2,355 (32.4)	1,362 (30.2)	0.046	1,289 (31.1)	1,270 (30.6)	0.010
Heart failure drugs, n (%)						
ACEis	1,553 (21.3)	979 (21.7)	0.010	907 (21.9)	897 (21.6)	0.006
ARBs	1,665 (22.9)	1,131 (25.1)	0.052	1,021 (24.6)	1,021 (24.6)	< 0.001
Beta blockers	4,220 (58.0)	2,540 (56.4)	0.032	2,306 (55.6)	2,342 (56.5)	0.018
Calcium channel blockers	2,400 (33.0)	1,385 (30.7)	0.048	1,290 (31.1)	1,286 (31)	0.002
Diuretics	4,880 (67.0)	2,751 (61.1)	0.125	2,554 (61.6)	2,579 (62.2)	0.012
Potassium sparing diuretics	1,619 (22.2)	935 (20.8)	0.036	855 (20.6)	864 (20.8)	0.005
ARNI	300 (4.1)	191 (4.2)	0.006	182 (4.4)	173 (4.2)	0.011
SGLT2i	588 (8.1)	387 (8.6)	0.019	347 (8.4)	345 (8.3)	0.002
Lipid-lowering medications, n (%)						
HMG CoA reductase inhibitors	3,050 (41.9)	1,887 (41.9)	< 0.001	1,705 (41.1)	1,734 (41.8)	0.014

ACEi, angiotensin-converting enzyme inhibitor; ARB, angiotensin receptor blocker; ARNI, angiotensin receptor-neprilysin inhibitor; SGLT2i, sodium-glucose cotransporter-2 inhibitor; Std Diff, standardized difference; ZD, zinc deficiency.

Standardized difference (Std diff) < 0.1 is considered a small difference.

After PSM, the ZD (*n* = 4,145) and control (*n* = 4,145) groups were well balanced in baseline characteristics, as shown by standardized differences <0.1. Their mean ages were comparable (63.5 ± 16.5 vs. 63.7 ± 16.2 years), as were sex distributions (56.5% vs. 56.6% female). Comorbidities such as type 2 diabetes, hypertension, overweight/obesity, and chronic kidney disease were also well matched between groups. Similarly, albumin levels, HbA1c, and eGFR showed satisfactory balance, ensuring comparability in clinical characteristics (standardized differences <0.1; Table 1).

### Primary and secondary outcomes

For the primary outcome (all-cause mortality), the ZD group was associated with a significantly higher cumulative incidence compared with the control group (HR, 1.46; 95% CI, 1.29–1.66; *p* < 0.001), reflecting incidence rates of 13.47 and 9.78 per 100 person-years, respectively (Table 2). The *E*-value for this association was 2.28 (95% lower confidence limit [LCL], 1.90), suggesting that only a relatively strong unmeasured confounder could fully explain this observed association.

**Table 2:** Primary and secondary outcomes between the zinc deficiency group and the control group.

Outcome	ZD group (*n* = 4,145)		Control group (*n* = 4,145)		HR (95% CI)	*P* value	*E*-value (95% LCL)
	Events (*n*)	Incidence rate per 100 person-years	Events (*n*)	Incidence rate per 100 person-years			
Primary outcome							
All-cause mortality	558	13.47	405	9.78	1.46 (1.29, 1.66)	<0.001	2.28 (1.90)
MACEs	658	15.89	481	11.61	1.46 (1.30, 1.64)	<0.001	2.28 (1.92)
MAKEs	652	15.74	460	11.11	1.51 (1.34, 1.70)	<0.001	2.39 (2.01)
Secondary outcomes							
All-cause hospitalization	2,102	50.75	1,894	45.72	1.24 (1.16, 1.32)	<0.001	1.59 (1.45)

MACE, major adverse cardiovascular event; MAKE, major adverse kidney event; ZD, zinc deficiency.

Similarly, for MACEs, the ZD group was linked to an elevated risk (HR, 1.46; 95% CI, 1.30–1.64; *p* < 0.001), with incidence rates of 15.89 and 11.61 per 100 person-years in the ZD and control groups, respectively. The *E*-value for this association was 2.28 (95% LCL, 1.92), reinforcing the robustness of this finding. For MAKEs, a higher risk was also observed in the ZD group (HR, 1.51; 95% CI, 1.34–1.70; p < 0.001), with incidence rates of 15.74 and 11.11 per 100 person-years, respectively. The *E*-value of 2.39 (95% LCL, 2.01) further indicates that substantial unmeasured confounding would be required to nullify this result.

**Figure 2:**
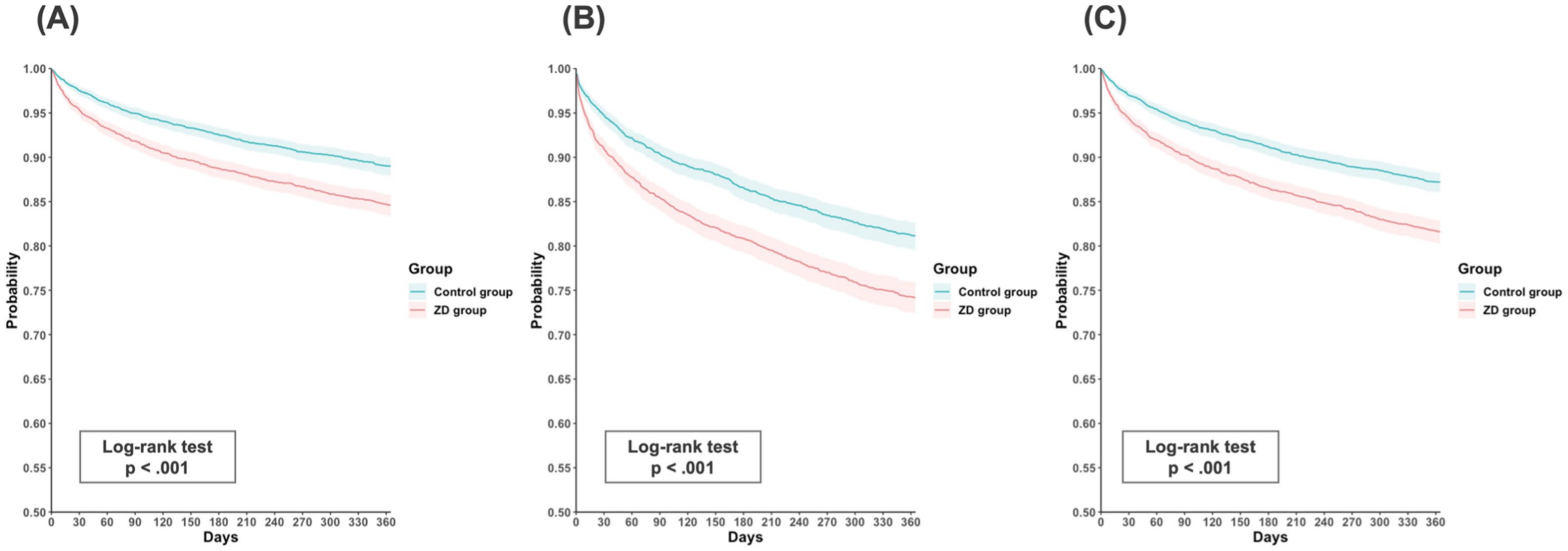
Kaplan–Meier time-to-event free curves of the primary outcomes: **(A)** all-cause mortality; **(B)** major adverse cardiovascular events; and **(C)** major adverse kidney events. ZD, zinc deficiency.

Regarding the secondary outcome of all-cause hospitalization, the ZD group was associated with a greater risk compared with the control group (HR, 1.24; 95% CI, 1.16–1.32; *p* < 0.001), with incidence rates of 50.75 and 45.72 per 100 person-years, respectively. The *E*-value of 1.59 (95% LCL, 1.45) suggests that moderate unmeasured confounding could potentially explain this association. Consistently, Kaplan–Meier curves demonstrated a higher cumulative incidence of all primary and secondary outcomes in the ZD group compared with the control group throughout the 1-year follow-up period (log-rank *p* < 0.001, Figures 2A–C).

### Stratified analysis

**Figure 3:**
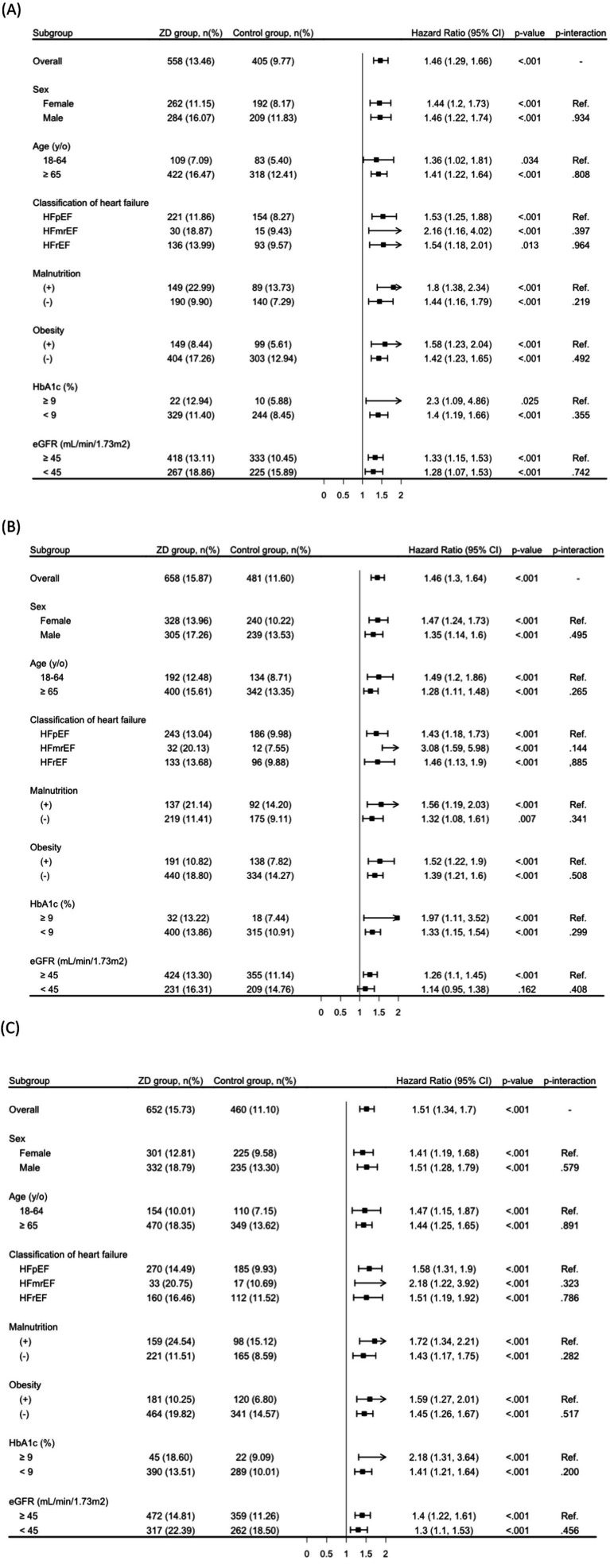
Stratified analysis of primary outcomes: **(A)** all-cause mortality; **(B)** major adverse cardiovascular events; and **(C)** major adverse kidney events. eGFR, estimated glomerular filtration rate; HbA1c, hemoglobin A1c; y/o, years old; ZD, zinc deficiency. Subgroup analyses were conducted using independently matched sub-cohorts based on propensity score matching within each stratum. Therefore, the number of patients and events may differ from those in the overall matched cohort.

Stratified analyses demonstrated that the association between ZD and significant risks of all-cause mortality, MACEs, and MAKEs remained consistent across most patient strata, including sex, age categories, presence of malnutrition, obesity, HF classifications, and HbA1c levels (Figures 3A–C). The only exception was observed for MACEs among patients with eGFR above 45 mL/min/1.73 m^2^, where the association did not reach statistical significance (Figure 3B). No significant interaction was observed across any of the subgroup variables (all P for interaction > 0.05), indicating a consistent association between zinc deficiency and adverse outcomes across strata (Figures 3A–C).

### Sensitivity analysis

The associations between ZD and adverse outcomes remained consistent across sensitivity analyses. When arrhythmias were excluded from the MACE definition, the HR for MACE in the ZD group was 1.42 (95% CI: 1.17–1.72; *p* < 0.001). Similarly, when cardiomyopathy was included as an additional component, the HR was 1.41 (95% CI: 1.19–1.68; p < 0.001). These findings further support the robustness of our main results (Supplementary Table S4).

After additional matching based on inflammatory and nutritional markers (CRP, copper, selenium, prealbumin, transferrin, and UACR), 2,478 patients remained in each group (ZD and control). As shown in Supplementary Table S5, baseline characteristics between the two groups were well balanced across all covariates. In this matched cohort, the association between ZD and all-cause mortality remained statistically significant (HR: 1.43, 95% CI: 1.20–1.69, p < 0.001), as did the associations with MACEs (HR: 1.17, 95% CI: 1.09–1.41), MAKEs (HR: 1.57, 95% CI: 1.08–2.29), and all-cause hospitalization (HR: 1.23, 95% CI: 1.15–1.33) (Supplementary Table S6).

## Discussion

In this large, multicenter retrospective cohort study of over 8,000 patients with HF, we found that ZD was associated with a significantly higher risk of all-cause mortality, MACE, MAKE, and increased all-cause hospitalization within 1 year of follow-up. These findings contribute important new evidence to the growing body of literature linking ZD to adverse clinical outcomes in HF and highlight the potential clinical relevance of maintaining optimal zinc levels in this high-risk population.

A salient observation from the study is the consistently higher hazard for all three primary outcomes—mortality, MACEs, and MAKEs—in patients with ZD compared to those with normal zinc levels, even after robust PSM. These results reinforce and extend earlier, smaller investigations that have implicated suboptimal zinc status in increased cardiovascular risk. For instance, a previous longitudinal study demonstrated that patients with HF and serum zinc levels below 62 μg/dL had elevated risks of cardiovascular and all-cause mortality (24). Similarly, a pilot study suggested that supplementation with intravenous zinc in selected patients led to partial improvement in LVEF (25), hinting at a possible therapeutic benefit. By leveraging a large-scale dataset derived from multiple healthcare organizations, our study provides a strong epidemiologic foundation indicating that ZD may be a critical, modifiable risk factor in HF management.

Multiple mechanistic pathways may underlie the observed increased risk associated with ZD. Zinc is integral to numerous enzymatic processes and regulatory pathways that maintain cardiovascular homeostasis. *In vitro* and animal studies have shown that zinc plays a key role in mediating antioxidant defenses, mitigating oxidative stress, and regulating inflammatory responses (6–12). A deficiency in zinc may intensify pro-inflammatory and pro-oxidative states, both of which can accelerate atherosclerosis, impair vascular endothelial function, and exacerbate myocardial remodeling. Additionally, zinc participates in cellular signaling pathways that modulate myocardial contractility, cellular apoptosis, and tissue repair (34–37). Consequently, suboptimal zinc status could predispose patients with HF to worsening cardiac function and increased susceptibility to arrhythmogenic events, thereby contributing to higher risks of MACEs and mortality (14).

Furthermore, prior work has shown that patients with HF are prone to hyperzincuria, especially when on diuretics and renin-angiotensin-aldosterone system inhibitors (15–19). Although these medications confer substantial benefits in HF, they may inadvertently lower systemic zinc levels over time. Similarly, when zinc is depleted, the kidney may be more susceptible to tubular damage, fibrosis, and hemodynamic strain, especially under the chronic congestion and reduced perfusion characteristic of HF (38–40). Additionally, older age, malnutrition, and malabsorption syndromes—prevalent in many HF populations—can further impair zinc status, amplifying the cardiorenal burden. By undermining the kidney’s capacity to handle physiological stress, sustain filtration, and mitigate pro-inflammatory responses, insufficient zinc can exacerbate the progression to end-stage kidney disease or dialysis dependence (14, 41).

In recent years, a growing number of systematic reviews have examined the relationship between zinc status and cardiovascular outcomes (7, 14). For example, Rosenblum et al. (14) conducted a systematic review highlighting the potential link between zinc deficiency and adverse prognosis in patients with HF. However, the current body of evidence remains limited, and the heterogeneity among existing studies has precluded the conduct of a robust meta-analysis. Our study contributes novel data that complement prior findings by adopting a large-scale, multicenter cohort design using standardized real-world electronic medical records and rigorous propensity score matching. This methodological approach allows for a more granular and contemporary assessment of zinc deficiency in patients with HF, with uniform outcome definitions and consistent follow-up durations. Importantly, our results may serve as a valuable contribution to future meta-analyses and help inform clinical risk stratification strategies. These findings reinforce the emerging evidence and underscore the potential clinical relevance of zinc assessment in the management of HF.

Our findings underscore the potential clinical importance of assessing zinc status in HF. While worldwide trends and prevalence of ZD have shown some stability with notable reductions in general populations, disease-specific populations present a different picture (42). In our study, the prevalence of ZD in patients with HF was 62%, which aligns with previous research showing 66% in patients with HF, while other conditions like diabetes showed lower rates at 23% (30). The burden of ZD extends to other chronic conditions as well - affecting 30–66% of patients undergoing dialysis and 50–70% of cancer patients (38, 41, 43–46). Given that ZD is relatively common and potentially correctable, systematic screening and early detection could offer a new avenue for risk stratification and intervention. This is particularly relevant given the consistent associations with not only mortality but also MACEs and MAKEs, suggesting that ZD may affect both cardiac and renal trajectories in HF. Intriguingly, we also found a higher risk of all-cause hospitalization among ZD patients. This may have important economic and healthcare resource implications, considering that HF is already among the most resource-intensive conditions globally. Optimizing zinc levels might conceivably help reduce the frequency of hospital admissions, although this hypothesis should be tested in prospective trials.

The strengths of our study include a large cohort of patients with HF and ZD sourced from TriNetX, a global repository of real-world data encompassing multiple institutions and countries. This broad dataset enhances the generalizability of our findings to a wider population. Additionally, our study incorporates various stratified analyses and demonstrates high *E*-values, further supporting the robustness of our results. To address confounding factors, we used an integrated PSM function, ensuring a balanced comparison of baseline characteristics, including demographics and comorbidities, between the two groups.

However, our study has some limitations. First, its observational design prevents us from establishing causality. Second, ZD may act as a mediator of more advanced disease severity or overall malnutrition rather than being a direct contributor to negative outcomes. Third, reliance on EMRs and administrative billing codes may introduce misclassification bias in both HF diagnosis and serum zinc level measurements. To mitigate this, we applied validated ICD-10-CM codes and LOINC codes for zinc testing. Fourth, we lacked data on dietary zinc intake, the use of over-the-counter zinc supplements, and the exact timing and dosage of prescription medications, all of which could influence the observed association between ZD and HF outcomes. Fifth, our study focused on 1-year outcomes, which, while clinically meaningful, may not fully capture the long-term effects of ZD on disease progression and survival. A longer observation period could provide additional insights into risk magnitude and other clinically relevant endpoints. Finally, due to the aggregate and de-identified nature of the TriNetX platform, we were unable to perform advanced model-based metrics such as net reclassification improvement (NRI) or integrated discrimination improvement (IDI) to formally evaluate the incremental predictive value of serum zinc as a biomarker. Future studies using patient-level data and comprehensive risk prediction modeling are warranted to further assess the prognostic utility of zinc in clinical practice.

## Conclusion

The study demonstrates that ZD is significantly associated with elevated risks of all-cause mortality, MACEs, MAKEs, and all-cause hospitalization in patients with HF. These associations persisted despite careful adjustment for known prognostic factors. Although observational in nature, our results suggest that assessing and correcting ZD may represent a valuable yet underrecognized approach to improving outcomes in HF. Future prospective studies and RCTs are warranted to determine whether targeted interventions aimed at maintaining adequate zinc status can tangibly benefit this population.

## Data share statement

Data described in the manuscript, code book, and analytic code will be made publicly and freely available without restriction at: https://trinetx.com.

## Data Availability

The original contributions presented in the study are included in the article/Supplementary material, further inquiries can be directed to the corresponding authors.
