# Supplementary material for: Mortality and cardiorenal outcomes among heart failure patients with zinc deficiency: a multicenter retrospective cohort study of 8,290 patients
Source: Front Nutr. 2025 Apr 28;12:1589907. doi: 10.3389/fnut.2025.1589907 (PMC12066520; doi:10.3389/fnut.2025.1589907)
Supplement: Supplementary file 1 [file Table_1.docx]

**Table S1.** Demographic, diagnostic, procedural, medication, visit, and laboratory codes utilized in the definition of the cohorts.

| **Category** | **Code** | **Description** |
| --- | --- | --- |
| **#1 Age at least 18 years old** | | |
| Demographics | Age | Age (at least 18 years) |
| **#2 Visited HCOs at least twice between Jan 01, 2010 and Jun, 31 2025** | | |
| Visit | Visit | Visit |
| **#3 Diagnosed with HF** | | |
| Diagnosis | UMLS:ICD10CM:I50 | Heart failure |
| **#4 Had a zinc test within 1 year before the diagnosis of DM** | | |
| Labs | LONIC:8245-3 | Zinc [Mass/volume] in Blood |
| Labs | LONIC:5763-8 | Zinc [Mass/volume] in Serum or Plasma |
| **#5 No MACEs and MAKEs events occurred before the index date** | | |
| Diagnosis | UMLS:ICD10CM:I21 | Acute myocardial infarction |
| Diagnosis | UMLS:ICD10CM:I22 | Subsequent ST elevation (STEMI) and non-ST elevation (NSTEMI) myocardial infarction |
| Diagnosis | UMLS:ICD10CM:I63 | Cerebral infarction |
| Diagnosis | UMLS:ICD10CM:I65 | Occlusion and stenosis of precerebral arteries, not resulting in cerebral infarction |
| Diagnosis | UMLS:ICD10CM:I66 | Occlusion and stenosis of cerebral arteries, not resulting in cerebral infarction |
| Diagnosis | UMLS:ICD10CM:I67.89 | Other cerebrovascular disease |
| Diagnosis | UMLS:ICD10CM:I61 | Nontraumatic intracerebral hemorrhage |
| Diagnosis | UMLS:ICD10CM:I62 | Other and unspecified nontraumatic Intracranial hemorrhage |
| Diagnosis | UMLS:ICD10CM:I47.0 | Re-entry ventricular arrhythmia |
| Diagnosis | UMLS:ICD10CM:I47.2 | Ventricular tachycardia |
| Diagnosis | UMLS:ICD10CM:I49.0 | Ventricular fibrillation and flutter |
| Diagnosis | UMLS:ICD10CM:I49.3 | Ventricular premature depolarization |
| Diagnosis | UMLS:ICD10CM:I46.2 | Cardiac arrest due to underlying cardiac condition |
| Diagnosis | UMLS:ICD10CM:I46.9 | Cardiac arrest, cause unspecified |
| Diagnosis | UMLS:ICD10CM:Z99.2 | Dependence on renal dialysis |
| Diagnosis | UMLS:ICD10CM:N18.6 | End stage renal disease |
| Diagnosis | UMLS:ICD9CM:39.95 | Hemodialysis |
| Procedure | UMLS:CPT:90945 | Dialysis procedure other than hemodialysis (eg, peritoneal dialysis, hemofiltration, or other continuous renal replacement therapies), with single evaluation by a physician or other qualified health care professional |
| Procedure | UMLS:CPT:1012740 | Dialysis Services and Procedures |
| Procedure | UMLS:CPT:1006747 | Hemodialysis Access, Intervascular Cannulation for Extracorporeal Circulation, or Shunt Insertion Procedures on Arteries and Veins |
| Procedure | UMLS:CPT:1012752 | Hemodialysis Procedures |
| Diagnosis | UMLS:ICD10CM:N18.6 | End stage renal disease |
| Labs | LONIC:8001 | Estimated glomerular filtration rate by Creatinine-based formula (MDRD) |
| **#6 Have a follow-up record after the index date** | | |
| Visit | Visit | Visit |
| Deceased | Deceased | Deceased |
| Diagnosis | UMLS:ICD10CM:R99 | lll-defined and unknown cause of mortality |

CPT, Current Procedural Terminology; ICD9CM, International Classification of Diseases, ninth Revision, Clinical Modification; ICD10CM, International Classification of Diseases, Tenth Revision, Clinical Modification; LONIC, Logical Observation Identifiers Names and Codes; NLM, National Library of Medicine; RXNORM, medical prescription normalized; UMLS, Unified Medical Language System

**Table S2.** Definitions of covariates coding used in this study.

| **Code** | **Description** |
| --- | --- |
| Age at Index | Age at Index |
| Male | Male |
| Female | Female |
| White | White |
| Black or African American | Black or African American |
| Unknown Race | Unknown Race |
| Asian | Asian |
| Other Race | Other Race |
| 9037 | Hemoglobin A1c/Hemoglobin.total in Blood |
| 8001 | Glomerular filtration rate/1.73 sq M.predicted [Volume Rate/Area] in Serum, Plasma or Blood by Creatinine-based formula (MDRD) |
| 9045 | Albumin [Mass/volume] in Serum, Plasma or Blood |
| 2003 | Left Ventricular Ejection Fraction |
| UMLS:ICD10CM:F17 | Nicotine dependence |
| UMLS:ICD10CM:F10 | Alcohol related disorders |
| UMLS:ICD10CM:E40-E46 | Malnutrition |
| UMLS:ICD10CM:E66 | Overweight and obesity |
| UMLS:ICD10CM:I10 | Essential (primary) hypertension |
| UMLS:ICD10CM:E78 | Disorders of lipoprotein metabolism and other lipidemias |
| UMLS:ICD10CM:I20-I25 | Ischemic heart diseases |
| UMLS:ICD10CM:I48 | Atrial fibrillation and flutter |
| UMLS:ICD10CM:I60-I69 | Cerebrovascular diseases |
| UMLS:ICD10CM:N18 | Chronic kidney disease |
| UMLS:ICD10CM:J40-J4A | Chronic lower respiratory diseases |
| UMLS:ICD10CM:K70-K77 | Diseases of liver |
| UMLS:ICD10CM:M32 | Systemic lupus erythematosus |
| UMLS:ICD10CM:C00-D49 | Neoplasms |
| RXNMORM:1656328 | Sacubitril |
| NLM:RXNORM:CV100 | Beta blockers |
| NLM:RXNORM:CV700 | Diuretics |
| NLM:RXNORM:CV800 | Ace inhibitors |
| NLM:RXNORM:CV805 | Angiotensin ii inhibitor |
| NLM:RXNORM:A10BK | Sodium-glucose co-transporter 2 (sglt2) inhibitors |
| NLM:RXNORM:CV704 | Potassium sparing/combinations diuretics |
| NLM:RXNORM:CV200 | Calcium channel blockers |
| NLM:RXNORM:C10AA | Hmg coa reductase inhibitors |

ICD10CM, International Classification of Diseases, Tenth Revision, Clinical Modification

NLM, National Library of Medicine

RXNORM, medical prescription normalized

UMLS, Unified Medical Language System

**Table S3.** Definitions of outcomes coding used in this study.

| **Code** | **Description** |
| --- | --- |
| **All-cause mortality** | |
| Deceased | Deceased |
| UMLS:ICD10CM:R99 | Ill-defined and unknown cause of mortality |
| **Major adverse cardiovascular events** | |
| UMLS:ICD10CM:I21 | Acute myocardial infarction |
| UMLS:ICD10CM:I22 | Subsequent ST elevation (STEMI) and non-ST elevation (NSTEMI) myocardial infarction |
| UMLS:ICD10CM:I63 | Cerebral infarction |
| UMLS:ICD10CM:I65 | Occlusion and stenosis of precerebral arteries, not resulting in cerebral infarction |
| UMLS:ICD10CM:I66 | Occlusion and stenosis of cerebral arteries, not resulting in cerebral infarction |
| UMLS:ICD10CM:I67.89 | Other cerebrovascular disease |
| UMLS:ICD10CM:I61 | Nontraumatic intracerebral hemorrhage |
| UMLS:ICD10CM:I62 | Other and unspecified nontraumatic Intracranial hemorrhage |
| UMLS:ICD10CM:I47.0 | Re-entry ventricular arrhythmia |
| UMLS:ICD10CM:I47.2 | Ventricular tachycardia |
| UMLS:ICD10CM:I49.0 | Ventricular fibrillation and flutter |
| UMLS:ICD10CM:I49.3 | Ventricular premature depolarization |
| UMLS:ICD10CM:I46.2 | Cardiac arrest due to underlying cardiac condition |
| UMLS:ICD10CM:I46.9 | Cardiac arrest, cause unspecified |
| Deceased | Deceased |
| UMLS:ICD10CM:R99 | Ill-defined and unknown cause of mortality |
| **Major adverse kidney events** | |
| UMLS:ICD10CM:Z99.2 | Dependence on renal dialysis |
| UMLS:ICD10CM:N18.6 | End stage renal disease |
| UMLS:ICD9CM:39.95 | Hemodialysis |
| UMLS:CPT:90945 | Dialysis procedure other than hemodialysis (eg, peritoneal dialysis, hemofiltration, or other continuous renal replacement therapies), with single evaluation by a physician or other qualified health care professional |
| UMLS:CPT:1012740 | Dialysis Services and Procedures |
| UMLS:CPT:1006747 | Hemodialysis Access, Intervascular Cannulation for Extracorporeal Circulation, or Shunt Insertion Procedures on Arteries and Veins |
| UMLS:CPT:1012752 | Hemodialysis Procedures |
| UMLS:ICD10CM:N18.6 | End stage renal disease |
| LONIC:8001 | Estimated glomerular filtration rate by Creatinine-based formula (MDRD) |
| Deceased | Deceased |
| UMLS:ICD10CM:R99 | Ill-defined and unknown cause of mortality |

CPT, Current Procedural Terminology; ICD9CM, International Classification of Diseases, ninth Revision, Clinical Modification; ICD10CM, International Classification of Diseases, Tenth Revision, Clinical Modification; NLM, National Library of Medicine; RXNORM, medical prescription normalized; UMLS, Unified Medical Language System

**Table S4.** Sensitivity analysis of different MACE definition.

| Outcomes | HR (95% CI) | P value |
| --- | --- | --- |
| Original model (MI + stoke + arrhythmia + cardiac arrest) | 1.46 (1.30,1.64) | <.001 |
| MI + stoke + cardiac arrest | 1.42 (1.17,1.72) | <.001 |
| MI + stoke + cardiac arrest + cardiomyopathy | 1.41 (1.19,1.68) | <.001 |

CI, confidence interval; MI, myocardial infarction; HR, hazard ratio

**Table S5**. Baseline characteristics of included subjects in the inflammation- and nutritional-matched analysis.

| Variables | Before matching | | | After matching | | |
| --- | --- | --- | --- | --- | --- | --- |
|  | ZD group  (n=4,363) | Control group  (n=2,757) | Std  diff | ZD group  (n=2,478) | Control group  (n=2,478) | Std  diff |
| Age at index, years | | | | | | |
| Mean ± SD | 66.1 (15.5) | 62.6 (16.0) | 0.226 | 63.9 (15.9) | 63.6 (15.9) | 0.020 |
| Sex, n (%) | | | | | | |
| Female | 2,350 (54.0) | 1,570 (56.9) | 0.060 | 1,392 (56.2) | 1,404 (56.7) | 0.010 |
| Male | 2,003 (46.0) | 1,187 (43.1) | 0.060 | 1,086 (43.8) | 1,074 (43.3) | 0.010 |
| Race, n (%) | | | | | | |
| White | 2,720 (62.5) | 1,659 (60.2) | 0.047 | 1,481 (59.8) | 1,485 (59.9) | 0.003 |
| Black or African American | 589 (13.5) | 444 (16.1) | 0.072 | 363 (14.6) | 381 (15.4) | 0.020 |
| Asian | 82 (1.9) | 47 (1.7) | 0.013 | 37 (1.5) | 41 (1.7) | 0.013 |
| Other race | 179 (4.1) | 117 (4.2) | 0.007 | 113 (4.6) | 107 (4.3) | 0.012 |
| Unknown race | 749 (17.2) | 469 (17) | 0.005 | 465 (18.8) | 443 (17.9) | 0.023 |
| Estimated glomerular filtration rate, mL/min/1.73m^2^ | | | | | | |
| Mean ± SD | 69.3 (38.9) | 72.3 (33.9) | 0.083 | 71.8 (37.2) | 71.8 (34.8) | 0.000 |
| ≤ 45, n (%) | 1,948 (44.8) | 872 (31.6) | 0.273 | 862 (34.8) | 846 (34.1) | 0.014 |
| Albumin, g/dL |  |  |  |  |  |  |
| Mean ± SD | 3.3 (0.7) | 3.8 (0.6) | 0.716 | 3.5 (0.7) | 3.7 (0.6) | 0.343 |
| ≤ 3.5, n (%) | 2,808 (64.5) | 1,151 (41.7) | 0.468 | 1,141 (46) | 1,148 (46.3) | 0.006 |
| Prealbumin, mg/dL |  |  |  |  |  |  |
| Mean ± SD | 14.8 (7.5) | 19 (8.2) | 0.538 | 15.9 (7.8) | 18.9 (8.2) | 0.369 |
| ≤ 18, n (%) | 419 (9.6) | 171 (6.2) | 0.127 | 161 (6.5) | 165 (6.7) | 0.007 |
| Transferrin, mg/dL |  |  |  |  |  |  |
| Mean ± SD | 181.2 (90.0) | 196.7 (100.2) | 0.162 | 197.9 (97.3) | 192.4 (101.2) | 0.056 |
| ≤ 200, n (%) | 545 (12.5) | 231 (8.4) | 0.136 | 225 (9.1) | 229 (9.2) | 0.006 |
| Urine albumin-to-creatinine ratio, mg/g |  |  |  |  |  |  |
| Mean ± SD | 1126.1 (4433.6) | 314.5 (749.2) | 0.255 | 728.1 (1454.5) | 354 (799.8) | 0.319 |
| ≥ 30, n (%) | 68 (1.6) | 49 (1.8) | 0.017 | 50 (2.0) | 45 (1.8) | 0.015 |
| C reactive protein, mg/L |  |  |  |  |  |  |
| Mean ± SD | 50.6 (72.3) | 30.3 (51.9) | 0.324 | 43.5 (68.6) | 31.2 (52.7) | 0.200 |
| ≥ 5, n (%) | 1,755 (40.3) | 839 (30.4) | 0.208 | 840 (33.9) | 814 (32.8) | 0.022 |
| Copper, ug/dL |  |  |  |  |  |  |
| Mean ± SD | 112.9 (45.5) | 115.3 (37.6) | 0.059 | 111.8 (43.5) | 115.9 (37.5) | 0.101 |
| ≤ 70, n (%) | 44 (1.0) | 29 (1.1) | 0.004 | 27 (1.1) | 27 (1.1) | 0.000 |
| Selenium, ng/mL |  |  |  |  |  |  |
| Mean ± SD | 121.1 (45.1) | 118.8 (34.6) | 0.057 | 123.7 (50.1) | 119.6 (33.2) | 0.096 |
| ≤ 70, n (%) | 10 (0.2) | 10 (0.4) | 0.024 | 10 (0.4) | 10 (0.4) | 0.000 |
| HbA1c, % | | | | | | |
| Mean ± SD | 6.3 (1.7) | 6.4 (1.5) | 0.058 | 6.4 (1.6) | 6.4 (1.5) | 0.027 |
| ≥ 9, n (%) | 280 (6.4) | 191 (6.9) | 0.020 | 159 (6.4) | 167 (6.7) | 0.013 |
| Left ventricular ejection fraction, % |  |  |  |  |  |  |
| Mean ± SD | 55.0 (15.2) | 53.8 (15.3) | 0.079 | 54.1 (15.5) | 53.8 (15.1) | 0.019 |
| < 40, n (%) | 145 (2.6) | 95 (2.6) | 0.003 | 88 (2.7) | 85 (2.6) | 0.006 |
| Comorbidities, n (%) |  |  |  |  |  |  |
| Hypertension | 2,731 (62.7) | 1,778 (64.5) | 0.036 | 1,567 (63.2) | 1,565 (63.2) | 0.002 |
| Hyperlipidemia | 2,072 (47.6) | 1,451 (52.6) | 0.101 | 1,237 (49.9) | 1,262 (50.9) | 0.020 |
| Overweight and obesity | 1,425 (32.7) | 1,150 (41.7) | 0.186 | 947 (38.2) | 958 (38.7) | 0.009 |
| Malnutrition | 982 (22.6) | 340 (12.3) | 0.272 | 325 (13.1) | 335 (13.5) | 0.012 |
| Type 2 diabetes mellitus | 1,623 (37.3) | 1,065 (38.6) | 0.028 | 927 (37.4) | 951 (38.4) | 0.020 |
| Nicotine dependence | 582 (13.4) | 301 (10.9) | 0.075 | 271 (10.9) | 276 (11.1) | 0.006 |
| Alcohol related disorders | 386 (8.9) | 144 (5.2) | 0.143 | 137 (5.5) | 140 (5.7) | 0.005 |
| Chronic lower respiratory diseases | 1,511 (34.7) | 903 (32.8) | 0.041 | 788 (31.8) | 818 (33.0) | 0.026 |
| Diseases of liver | 1,026 (23.6) | 456 (16.5) | 0.176 | 440 (17.8) | 445 (18.0) | 0.005 |
| Chronic kidney disease | 1,363 (31.3) | 628 (22.8) | 0.193 | 594 (24.0) | 602 (24.3) | 0.008 |
| Cerebrovascular diseases | 432 (9.9) | 210 (7.6) | 0.082 | 197 (8.0) | 196 (7.9) | 0.001 |
| Atrial fibrillation and flutter | 1,382 (31.7) | 748 (27.1) | 0.101 | 659 (26.6) | 703 (28.4) | 0.040 |
| Ischemic heart diseases | 1,651 (37.9) | 987 (35.8) | 0.044 | 867 (35.0) | 893 (36.0) | 0.022 |
| Systemic lupus erythematosus | 70 (1.6) | 45 (1.6) | 0.002 | 46 (1.9) | 40 (1.6) | 0.019 |
| Neoplasms | 1,409 (32.4) | 806 (29.2) | 0.068 | 768 (31) | 755 (30.5) | 0.011 |
| Heart failure drugs, n (%) |  |  |  |  |  |  |
| ACEis | 934 (21.5) | 613 (22.2) | 0.019 | 559 (22.6) | 550 (22.2) | 0.009 |
| ARBs | 985 (22.6) | 729 (26.4) | 0.089 | 623 (25.1) | 617 (24.9) | 0.006 |
| Beta blockers | 2,486 (57.1) | 1,536 (55.7) | 0.028 | 1,367 (55.2) | 1,368 (55.2) | 0.001 |
| Calcium channel blockers | 1,340 (30.8) | 803 (29.1) | 0.036 | 752 (30.3) | 725 (29.3) | 0.024 |
| Diuretics | 2,943 (67.6) | 1,737 (63) | 0.097 | 1,572 (63.4) | 1,567 (63.2) | 0.004 |
| Potassium sparing diuretics | 1,047 (24.1) | 649 (23.5) | 0.012 | 575 (23.2) | 578 (23.3) | 0.003 |
| ARNI | 188 (4.3) | 123 (4.5) | 0.007 | 112 (4.5) | 107 (4.3) | 0.010 |
| SGLT2i | 388 (8.9) | 283 (10.3) | 0.046 | 254 (10.3) | 246 (9.9) | 0.011 |
| Lipid-lowering medications, n (%) |  |  |  |  |  |  |
| HMG CoA reductase inhibitors | 1,848 (42.5) | 1,177 (42.7) | 0.005 | 1,052 (42.5) | 1,055 (42.6) | 0.002 |

ACEi: angiotensin-converting enzyme inhibitor; ARB: angiotensin receptor blocker; ARNI: angiotensin receptor-neprilysin inhibitor; SGLT2i: sodium-glucose cotransporter-2 inhibitor; Std Diff: standardized difference; ZD: zinc deficiency

Standardized difference (Std diff) < 0.1 is considered a small difference.

**Table S6.** Primary and secondary outcomes between the zinc deficiency group and the control group after matching additional inflammatory and nutritional markers.

| Outcome | ZD group (n = 2,478) | | Control group (n = 2,478) | | HR (95% CI) | *P* value | E-value (95% LCL) |
| --- | --- | --- | --- | --- | --- | --- | --- |
|  | Events (n) | Incidence rate  per 100 person-years | Events (n) | Incidence rate  per 100 person-years |  |  |  |
| Primary outcome |  |  |  |  |  |  |  |
| All-cause mortality | 307 | 12.4 | 230 | 9.3 | 1.43 (1.20,1.69) | <.001 | 2.21 (1.69) |
| MACEs | 212 | 8.6 | 180 | 7.3 | 1.17 (1.09,1.41) | 0.024 | 1.47 (1.32) |
| MAKEs | 67 | 2.7 | 46 | 1.9 | 1.57 (1.08,2.29) | 0.017 | 2.52 (1.37) |
| Secondary outcomes |  |  |  |  |  |  |  |
| All-cause hospitalization | 1,594 | 64.4 | 1,445 | 58.4 | 1.23 (1.15,1.33) | <.001 | 1.58 (1.44) |

MACE: major adverse cardiovascular event; MAKE: major adverse kidney event; ZD: zinc deficiency
